# Understanding and exploring the diversity of soil microorganisms in tea (*Camellia sinensis*) gardens: toward sustainable tea production

**DOI:** 10.3389/fmicb.2024.1379879

**Published:** 2024-04-12

**Authors:** Motunrayo Y. Jibola-Shittu, Zhiang Heng, Nemat O. Keyhani, Yuxiao Dang, Ruiya Chen, Sen Liu, Yongsheng Lin, Pengyu Lai, Jinhui Chen, Chenjie Yang, Weibin Zhang, Huajun Lv, Ziyi Wu, Shuaishuai Huang, Pengxi Cao, Lin Tian, Zhenxing Qiu, Xiaoyan Zhang, Xiayu Guan, Junzhi Qiu

**Affiliations:** ^1^Key Lab of Biopesticide and Chemical Biology, Ministry of Education, State Key Laboratory of Ecological Pest Control for Fujian and Taiwan Crops, College of Life Sciences, Fujian Agriculture and Forestry University, Fuzhou, China; ^2^Department of Biological Sciences, University of Illinois, Chicago, IL, United States; ^3^School of Ecology and Environment, Tibet University, Lhasa, China; ^4^Tibet Plateau Institute of Biology, Lhasa, China; ^5^Fuzhou Technology and Business University, Fuzhou, Fujian, China; ^6^College of Horticulture, Fujian Agriculture and Forestry University, Fuzhou, Fujian, China

**Keywords:** microbial communities, soil health, soil microorganisms, tea gardens, tea plant

## Abstract

Leaves of *Camellia sinensis* plants are used to produce tea, one of the most consumed beverages worldwide, containing a wide variety of bioactive compounds that help to promote human health. Tea cultivation is economically important, and its sustainable production can have significant consequences in providing agricultural opportunities and lowering extreme poverty. Soil parameters are well known to affect the quality of the resultant leaves and consequently, the understanding of the diversity and functions of soil microorganisms in tea gardens will provide insight to harnessing soil microbial communities to improve tea yield and quality. Current analyses indicate that tea garden soils possess a rich composition of diverse microorganisms (bacteria and fungi) of which the bacterial Proteobacteria, Actinobacteria, Acidobacteria, Firmicutes and Chloroflexi and fungal Ascomycota, Basidiomycota, Glomeromycota are the prominent groups. When optimized, these microbes’ function in keeping garden soil ecosystems balanced by acting on nutrient cycling processes, biofertilizers, biocontrol of pests and pathogens, and bioremediation of persistent organic chemicals. Here, we summarize research on the activities of (tea garden) soil microorganisms as biofertilizers, biological control agents and as bioremediators to improve soil health and consequently, tea yield and quality, focusing mainly on bacterial and fungal members. Recent advances in molecular techniques that characterize the diverse microorganisms in tea gardens are examined. In terms of viruses there is a paucity of information regarding any beneficial functions of soil viruses in tea gardens, although in some instances insect pathogenic viruses have been used to control tea pests. The potential of soil microorganisms is reported here, as well as recent techniques used to study microbial diversity and their genetic manipulation, aimed at improving the yield and quality of tea plants for sustainable production.

## Graphical abstract

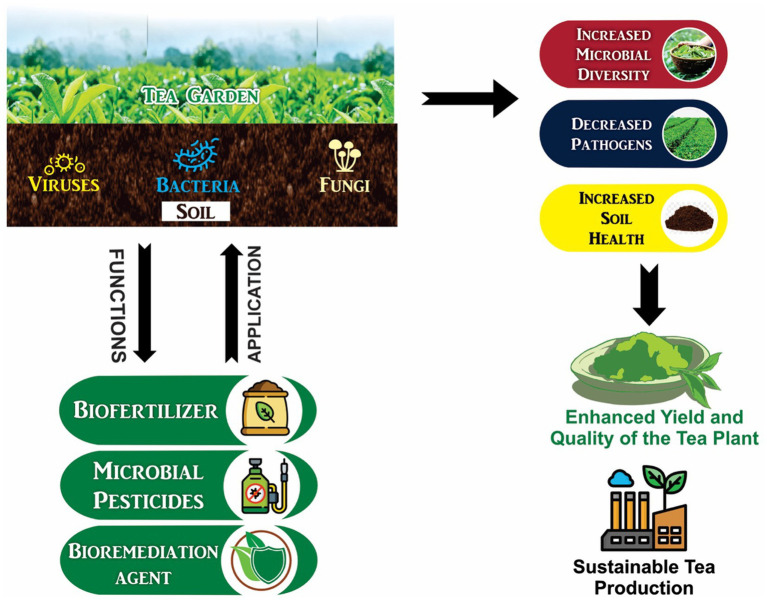

## 1 Introduction

*Camellia sinensis* commonly referred to as the “tea plant” is an economically important crop, belonging to the family Thaeceae (Pandey et al., 2021). As a brewed beverage, it has been consumed for at least several millennia, with initial indications that leaves were first eaten raw or added to soups followed by fermentation and chewing of the leaves. Subsequently, it was turned into a beverage by mixing fresh or cured leaves with hot or boiling water, with early written description of tea drinking dating to at least the 3^rd^ century A.D. in China. Modern tea consumption is second only to water, and tea production is dominated by India/Sri Lanka and China, with the latter accounting for about half of the tea produced in our world today (Xu et al., 2018, 2022). Tea is cultivated predominantly in tropical and subtropical regions of the world (Hazra et al., 2019), and global tea consumption is estimated to have increased ~43% from 2005 to 2020 (FAOSTAT, 2022). This is likely due in part to the many putative beneficial health and medicinal values of tea due to its wide range of bioactive constituents. One well known component of tea is caffeine, which can act as a stimulant increasing alertness, with levels of caffeine affected by leaf harvest time and various forms of post-harvest processing. In addition, tea contains a range of secondary metabolites, some of which may possess antioxidant, digestive, (putative) antimicrobial, and/or other health promoting benefits to the consumers (Pokharel et al., 2023; Ramphinwa et al., 2023). Aside from caffeine, potential bioactive compounds include polyphenols (e.g., flavonoids and catechins) as well as xanthines such as theobromine and theophylline. Tea consumption has been linked cancer-prevention, treatment of various cardiovascular problems, and improved circulation potentially due to the variety of polyphenols, antioxidants, and other compounds they possess, although definitive clinical data is lacking (Perez-Burillo et al., 2019; Abe and Inoue, 2021; Bag et al., 2022). Tea consumption also provides important comfort and social interactions in many societies worldwide. Nevertheless, the demand for tea is projected to see a continuous increase (Xi et al., 2023). Therefore, to effectively cater for the rising demand for tea, research should be aimed at the development of ecologically friendly and sustainable approaches to improving the quality and yield of tea.

*Camellia sinensis* is native to East Asia, with a purported origin along the Irrawady River, spreading into present day southeast China, India, and later to Sri Lanka. Two main cultivated varieties of traditional tea have thus far been described, *C. sinensis* var. *sinensis* and *C. sinensis var. assamica*, that are separated into distinct clades and likely have different parentages. However, *C. sinensis* var*. assamica* can be separated into two subtypes, namely, Southern Yunnan Assam (China) and Indian Assam (India), which although may have originated from the same parent, appears to represent two independent domestication events. Furthermore, some subvarieties appear to have undergone hybridization with closely related species such as *C. taliensis* and *C. pubicosta* (Mukhopadhyay and Mondal, 2017; Auria et al., 2022). In addition to the occurrence of different regional varieties, tea is further classified post-harvest via the different means and methods for processing of the leaves that result in significant differences in the final consumed product (Auria et al., 2022). These different post-harvest processing methods result in the commonly referred to black, green, white, oolong, dark, and yellow teas (among others), and involve a series of steps that can include, depending upon the final outcome, the following: (picking), (i) withering—drying of the leaves; under sun for darker teas, in a cool ventilated room for lighter teas, (ii) bruising—crushing, shaking, rolling, and/or other forms of manipulating the leaves; mainly for darker teas, (iii) oxidation—exposure leaves to air for different period of time; darkens teas depending upon time, (iv) heating—after oxidation, leaves are heated to stop oxidation process, also referred to as “fixation,” (v) yellowing—light heating in closed chamber, (vi) fermentation—leaves are allowed to ferment for a period of time; results in increase in sweetness, (vii) drying—remove moisture via baking, sun and/or air-drying, (viii) sorting and shaping—stems, seeds, and impurities are removed and the tea “shaped” into various forms, e.g., bricks, circles, etc., for aging and/or storage. Each “type” of tea has its own sequence of specific steps as outlined above, but not all. Thus, “black” tea involves withering, bruising, oxidation, shaping, and drying, “white” tea (freshly picked leaf buds): only withering, heating, shaping, and drying (with white tea often considered the least “processed” of the final tea forms). In addition, many of these steps can have important production differences with respect to leaf treatment for any given step in terms of time, temperature, and other conditions that can result in significant differences in the final products even if all are considered “black” teas (Auria et al., 2022; Aaqil et al., 2023).

Although post-harvest processing is relatively well-described, the soil support used to produce tea has been less studied despite anecdotal and regional recognition that variations in soil “quality” affects plant growth and subsequent leaf quality and production. It is well known that soil microorganisms (bacteria, fungi, and viruses, e.g., the soil microbiome) functions in mediating soil health, and subsequent plant growth and crop yield (Wang et al., 2017; Gu et al., 2019). These effects can be positive or negative with respect to plant health, with beneficial microbes helping to: (i) mobilize otherwise (plant) recalcitrant nutrients, particularly nitrogen and phosphorus, to the plant (often in exchange for carbon), (ii) facilitate plant resistance to abiotic stress including temperature and drought, and (iii) protect plants from infection and disease. In contrast, harmful microbes, e.g., biotrophic and necrotrophic plant pathogens can cause disease, and competition with some microbes may decrease overall plant access to nutrients. Overall, however, the diversity of soil microbial communities can serve as an indicator of soil fertility and soil health (Gui et al., 2022), and poor soil (in terms of mediating plat health) typically showing a reduction in soil microbial community diversity, which can then result in adverse effects on the sustainable utilization of soil resources (Chen et al., 2015). Therefore, maintaining the diversity of soil microbial communities, with an emphasis on identifying and enriching for beneficial microbes, can exert a significant impact on managing soil organic carbon and nutrient availability to plants thus increasing the sustainability of agricultural ecosystems (Bertola et al., 2021; Chauhan et al., 2023), particularly given that several studies have shown that soil microorganisms can have important positive effects on plant growth, plant health, resistance to abiotic stress, and overall agricultural productivity (Trivedi et al., 2020; Qiao et al., 2024). Due to the economic importance of tea plants, it is valuable to build models integrating the nature of soil microorganisms and the vital functions they perform with respect to tea cultivation. Here, we review current information concerning the diversity and potential functions of soil microbial communities in tea gardens, to provide insights into less reported factors that could be explored to improve tea cultivation by examining and potentially manipulating the diversity of soil microorganisms. We also highlight the various techniques used for studying soil microbial diversity within tea gardens. The identification of the diverse groups of soil microorganisms as well as their potential functions will help in meeting the growing demand for the sustainable production of tea plants with high quality and yield.

## 2 Overview of soil microbiome activities and recent approaches to soil microbiome studies in tea gardens

Soil microbial communities are an essential part of the soil ecosystem, consisting of diverse fungi, bacteria, and viruses (Naylor et al., 2022). Generally, soil microorganisms are involved in key processes in the soil ecosystem; they mediate organic matter decomposition, nutrient cycling, and gaseous fluxes, and impact soil geochemistry including pH, trace metal and other element content, and phosphorus availability, all of which have resultant effects on plant nutrient availability and resistance to stress (Bastida et al., 2021; Hartmann and Six, 2022). Although carbon is gained via photosynthesis, other primary nutrients such as nitrogen, phosphorus, sulphur and potassium, required for plant growth and development, are made available for plant uptake through cycling and transformation processes in the soil. These processes are actively mediated by soil microorganisms, and the availability of these nutrients for plant uptake is a determinant of soil fertility (Basu et al., 2021; Nabi, 2023). Highly fertile soils often exhibit increased bacterial diversity, predominantly those belonging to the Proteobacteria, Nitrospira, Chloroflexi, and Bacteroidetes, in addition to demonstrating enhanced functions such as nitrate reduction, ammonia oxidation and aromatic compound degradation in contrast with low fertile soils (Da Costa et al., 2024). For many crops, bacteria of prominent groups involved in nitrogen-cycling processes and maintaining soil nitrogen balance are indicative of soil fertility, and correlate with crop yield (Bayer et al., 2020; Hayatsu et al., 2021).

In addition, the soil microbiome can participate in the bioremediation of pollutants, heavy metals, and other compounds that can adversely affect plant health or could otherwise affect the quality (and human health safety) of the tea leaves (Bastida et al., 2021; Phillippot et al., 2023). Nitrogen which is a key element involved in the growth and quality tea plants leaves (Ma et al., 2021), is usually recycled by nitrogen-metabolizing microorganisms present in the rhizosphere, providing for enhanced nitrogen absorption of plant usable forms (NO^−^_3_ and NH^+^_4_) (Liu et al., 2017, 2022) that can then impact the growth and yield of tea. In tea root systems, ammonia is converted into theanine, a non-protein amino acid that adds a distinct rich flavor to tea as the root absorbs such nitrogen sources from the soil (Cheng et al., 2017; Dong et al., 2019). Theanine is then transported to the leaves and young shoots which are harvested during tea production (Zhang et al., 2023). Within this context, a consortium of nitrogen-metabolizing soil microorganisms predominantly belonging to the Proteobacteria and other phyla such as the Actinobacteria, Firmicutes, Chloroflexi, and Armatimonadetes have been reported in tea roots to help enhance ammonia uptake and subsequent theanine synthesis, thus contributing to the taste and quality of tea leaves (Xin et al., 2024).

Tea cultivation is unique in that an important number of tea “gardens” or areas of tea cultivation, have existed, i.e., been continuously cultivated with *C. sinensis*, for significant periods of times (generations or even more in some instances). With such relatively continuous cultivation in specific areas, it is likely strong co-interactions between the tea plants and resident microorganisms in the soil have developed, including potentially unique co-adaptations. However, many areas of tea cultivation have also had significant inputs (fertilizer, pesticides, even soil) from other areas that can impact the diversity of both beneficial and harmful (to the plant) microbes (Fu et al., 2021). In addition, tea gardens are faced with a variety of challenges from insect pests and microbial diseases, many of which are vectored by insects (Zhang X. et al., 2022). As suggested, to achieve high yield, significant amounts of fertilizers and pesticides are used in some tea gardens which can, after long term use, cause a decline in soil microbial diversity and hence results in a negative environmental impact (Wang et al., 2020). As tea gardens are usually found on elevated plains, application of chemical fertilizers and pesticides can easily run-off into downstream water bodies, causing eutrophication and pollution of the water (Xie et al., 2021).

Several studies on tea garden soils have revealed the presence of a vast array of microorganisms which are linked to the quality of tea produced from the soil (Fu et al., 2021; Kui et al., 2021; Bag et al., 2022), particularly as soil microbial communities participate in promoting soil health and suppressing plant pathogens (Wu et al., 2023). Based on culture-dependent approach and molecular identification of bacterial isolates through 16S rRNA gene sequencing, keystone bacteria genera such as *Bacillus, Burkholderia, Serratia* and *Arthrobacter* (Table 1) have been reported to display a wide range of growth promoting activities. For example, the phosphorus solubilizing abilities in *Burkholderia* and *Bacillus* isolated from rhizospheric soil samples of tea gardens in West Bengal, India have been characterized (Panda et al., 2017), and isolation of a *Burkholderia pyrrocinia* strain from the tea rhizosphere contributing to abiotic stress tolerance and possessing plant growth promoting activities through phosphorus solubilization and production of phytohormones has also been reported (Han et al., 2021). In addition, a biocontrol strain of *Serratia marcescens* was reportedly isolated from tea rhizosphere displayed significant biocontrol efficacy against fungal root pathogens of tea through the production of hydrolytic enzymes such as chitinase, protease, lipase and cellulase, as well as the production of antibiotics (Dhar Purkayastha et al., 2018). These studies indicate that knowledge concerning the microbial ecosystem of tea soils and the processes by which they help improve tea quality, should be considered an important avenue for further exploration aimed toward enhancing nutrient availability in tea gardens and the resultant yield and quality of tea plants.

**Table 1:** Some key microbial species associated with soil ecosystem of tea gardens.

Microbial species	Bacteria phyla	Function	References
*Arthrobacter* sp. MT436081	Actinobacteria	Phosphorus solubilizing, biocontrol	Bhattacharyya et al. (2020)
*Bacillus firmus* HNS012 *Bacillus firmus* UST000620-011	Firmicutes	Phosphorus solubilizing	Panda et al. (2017)
*Bacillus megaterium* MT436102	Firmicutes	Phosphorus solubilizing, ammonia production, protease production, cellulase production	Bhattacharyya et al. (2020)
*Bacillus pseudomycoides* SN29 (KJ767523)	Firmicutes	Ammonia production, phosphorus solubilizing, production of phytohormones	Dutta et al. (2015)
*Bacillus velezensis* MT436088 *Bacillus velezensis* MT436091	Firmicutes	Phosphorus solubilizing, ammonia production, protease production	Bhattacharyya et al. (2020)
*Brevibacillus agri* KX373961	Firmicutes	Phosphorus solubilizing, ammonia production, biocontrol, production of phytohormones	Dutta and Thakur (2017)
*Brevibacterium sediminis* A6	Actinobacteria	Phosphate solubilizing, ammonia production, biocontrol	Chopra et al. (2020a)
*Burkholderia arboris* R24201	Proteobacteria	Phosphorus solubilizing	Panda et al. (2017)
*Burkholderia cepacia* ATCC177759 *Burkholderia cepacia* ATCC 35254	Proteobacteria	Phosphorus solubilizing	Panda et al. (2017)
*Burkholderia pyrrocinia* P10 (DSM 10685^T^)	Proteobacteria	Abiotic stress tolerance	Han et al. (2021)
*Burkholderia* sp. J62 *Burkholderia* sp. YXA1-13	Proteobacteria	Phosphorus solubilizing	Panda et al. (2017)
*Burkholderia* sp. TT6 (KJ767524)	Proteobacteria	Ammonia production, phosphorus solubilizing, production of phytohormones	Dutta et al. (2015)
*Burkholderia vietnamiensis* TVV70	Proteobacteria	Phosphorus solubilizing	Panda et al. (2017)
*Claroideoglomus* sp.	Glomeromycota	Nutrient uptake and Leaf food quality	Wu et al. (2019) and Shao et al. (2019)
*Enterobacter lignolyticus* TG1 (KJ767522)	Proteobacteria	Ammonia production, phosphorus solubilizing, production of phytohormones	Dutta et al. (2015)
*Enterobacter* sp. KX373977	Proteobacteria	Phosphorus solubilizing, ammonia production, biocontrol, production of phytohormones	Dutta and Thakur (2017)
*Glomus* sp.	Glomeromycota	Growth promoting activities	Wu et al. (2019); Sun et al. (2020)
*Glomus viscosum*	Glomeromycota	Nutrient uptake	Wu et al. (2019)
*Pseudomonas aeruginosa* KH45 (KJ767521)	Proteobacteria	Ammonia production, phosphorus solubilizing, production of phytohormones	Dutta et al. (2015)
*Serratia marcescens*	Proteobacteria	Phosphorus solubilizing, production of phytohormones. Biocontrol	Panda et al. (2017)
*Serratia marcescens* ETR17	Proteobacteria	Biocontrol, Phosphorus solubilizing, production of phytohormones	Dhar Purkayastha et al. (2018)

Exploring soil microbial diversity is beneficial for the development of agricultural ecosystems as well as testing the effectiveness of restoration measures (Deltedesco et al., 2020). For tea gardens such studies can help in understanding the functions of microorganisms and in harnessing them for better productivity of tea. Recently, the development of culture-independent metagenomics techniques, has contributed greatly to mapping soil microbial phylogeny (Su et al., 2017). The development and refinement of molecular techniques such as the high-throughput sequencing have greatly promoted the study and understanding of diversity and interactions of soil microorganisms (Wei et al., 2018; Song et al., 2023) and has given rise to a significant improvement in terms of both rDNA homology and descriptions of biosynthetic pathways (Wei et al., 2018). Consequently, metagenomic analyses display strong reliability and convenience for characterizing root-associated microorganisms (Bhattacharyya et al., 2016; Busby et al., 2017), and is being applied to characterize soil microbial diversity by directly capturing total soil microbial DNA (Sharma and Kaur, 2021; Parihar et al., 2022) providing a window into functional aspects of soil microorganisms (Wei et al., 2018).

Next,-generation sequencing technologies have also led to insights into various environmental factors contributing to soil microbial diversity (Egidi et al., 2019). As part of this, the majority of microbial species assigned to “RNA similarity groups” can help provide a deeper understanding of the changes in diversity and composition of soil microbial communities (Chen et al., 2017). Similarly, high through-put sequencing technologies have enabled microbiologists to sequence amplified gene markers (e.g., 16S ribosomal RNA), to determine phylogenetic and functional diversity profiles of soil microbial communities (Wu et al., 2015). High-throughput sequencing and molecular ecology network (MEN) analyses have been used to investigate soil microbial diversity, community structure, composition, and interaction networks of tea plantations, revealing the diversity, and dominance of Proteobacteria, Acidobacteria, and Chloroflexi in all tea plantation samples under different management practices (Tan et al., 2019). Although still limited, targeted gene manipulation, e.g., use of CRISPR/Cas technologies has been applied to alter the expression of genes, study genetic diversity, and or produce modified microorganisms, and/or transfer of genes have been applied to tea cultivation research (Bag et al., 2022).

### 2.1 Fungal communities in tea gardens soils

Fungi are important drivers in soil ecosystems (Francioli et al., 2020); a rich fungal diversity in tea garden soils may help maintain healthy ecological functioning, including by facilitating nutrient cycling, organic matter decomposition, and plant productivity (Ma et al., 2022). In addition, (beneficial) fungi can play important roles in suppressing the activities of (microbial) plant pathogens present in the soil (Bollmann-Giolai et al., 2022). This latter function can be due to a variety of factors including excluding plant pathogen competitors to the production of certain metabolites targeting pathogenic microbes to stimulating plant antimicrobial defenses, thus suppressing tea pathogens, and enhancing tea yield and quality. Xu et al. (2022) have reported that fungal taxa that colonized tea shoots significantly inhibited fungal pathogens. These included fungal taxa corresponding to *Myriangium* and *Mortierella* which have been demonstrated to have plant growth-promoting abilities (Ozimek and Hanaka, 2021). Thus, these abilities may be linked to their ubiquity and potentials to protect plants against pathogens. Zheng et al. (2023) investigated the response of soil microbial communities and functions to long-term tea (*C. sinensis*) planting in a subtropical region and reported the relative abundance of fungal community in tea gardens to be largely dominated by Ascomycota (38.63–55.27%), Basidiomycota (19.45–39.13%), Mortierellomycota (1.8–10.1%), and Rozellomycota (0.12–7.41%). Similarly, Ma et al. (2022) in a study conducted on soils of tea plantations revealed that fungal community predominantly consisted of Ascomycota (44.7%), Mortierellomycota (17.7%) and Basidiomycota (11.4%) and accounted for 73.8% of total composition of fungal communities.

Earlier reports indicated that fungal communities in several tea gardens at the genus level are dominated primarily by *Saitozyma* (Ma et al., 2022; Wang et al., 2023). Members of the *Saitozyma,* have been shown to account for ~30% of the sequences in tea garden soils in the Southeast Asia region, followed by *Mortierella* (20%) and *Pseudogymnoascus* (10%) (Yan P. et al., 2022). Basidiomycota, such as *Saitozyma, Russula* and *Hygrocybe* commonly found in tea gardens, are well known to colonize lignin-rich surfaces and likely play significant roles in the degradation of lignin-rich plant litters (Guo et al., 2018; Kui et al., 2021), transforming these substrates to provide carbon and nitrogen as well as other nutrients for plant growth (Li et al., 2020). In addition, *Penicillium*, *Trichoderma* and *Pseudogymnoascus* are prominent members of the Ascomycota predominantly found in tea gardens (Kui et al., 2021; Wang et al., 2021; Yan L. et al., 2022). The abundance of the Ascomycota among soil fungal communities in tea gardens is perhaps because Ascomycota have been able to successfully evolve mechanisms to dominate soils globally (Egidi et al., 2019). These abilities of the Ascomycota include stress tolerance and production of secondary metabolites which can inhibit other microorganisms (Chen et al., 2017). Moreover, Ascomycota have been known to produce a wide range of antimicrobial agents, which can be advantageous to the protection of plants against pathogens.

The rhizosphere of tea gardens in Southeast Asia have been shown to contain a rich community of Glomeromycota, that include arbuscular mycorrhizal fungi (AMF), such as *Claroideoglomus, Acaulospora, Rhizophagus* and *Glomus* species. These fungi often colonize and form symbiotic relationships with the roots of tea plants (Bag et al., 2022; Zhang X. et al., 2022). This relationship with tea plant roots likely contributes to the ability of tea plants to thrive successfully for many years even under adverse environmental conditions that can include drought, salinity, and temperature. In particular, various AMF are known to provide host plants with essential mineral elements, confer resistance to pests, diseases and abiotic stress and promote plant health (Almario et al., 2022; Zhang X. et al., 2022). AMF members of the *Glomus, Acaulospora* and *Gigaspora* genera have all been reported in cultivated tea lands, e.g., in India and various locations of China (Sharma et al., 2013; Ji et al., 2022; Zhang Z. et al., 2022).

### 2.2 Bacterial communities in soils of tea gardens

Bacterial communities are diverse and perform numerous functions in soils. Kui et al. (2021), through the direct extraction of total soil DNA from soil samples and sequencing using high through-put 16S rRNA and internal transcribed spacer amplicon sequencing techniques, characterized the soil microbiome in ancient tea plantations in Southwest region, China, identifiying Acidobacteria, Actinobacteria, and Proteobacteria phyla as the dominant bacterial community. As tea farming often occurs in one place over many generations and sometimes hundreds of years the dominance of Acidobacteria may indicate their importance in key ecological processes such as regulation of biogeochemical cycles and growth promoting activities (Kalam et al., 2020) in tea gardens. The relative abundance of Acidobacteria in tea garden soils is linked to increased age of tea plants (Wang et al., 2019). The long-term use of pesticides and fertilizers which contributes to the acidification of tea soil enables the Acidobacteria to thrive through many mechanisms they have developed. These mechanisms are genetically controlled and include acid tolerance, secondary metabolites, nitrogen metabolism, exopolysaccharide synthesis, hopanoids synthesis, siderophore synthesis (Kalam et al., 2020; Yadav et al., 2021).

Furthermore, using Illumina Miseq sequencing of the 16S rRNA targeting rhizospheric soil bacteria, Zi et al. (2020) found the bacterial community to be dominated by Proteobacteria, Acidobacteria and Actinobacteria with the relative abundance of 43.12, 21.61, and 14.84%, respectively, in Southwest tea cultivation region of China. The dominance of Proteobacteria in soils of tea gardens may be linked to their functioning in carbon and nitrogen cycling because these bacteria are known to be involved in ammonia oxidation and nitrification (Zhang Z. et al., 2022; Wang et al., 2023). Besides, the use of nitrogen-based fertilizers to increase yield in tea gardens could be responsible for the abundance of Proteobacteria in soil because they are actively involved in nitrogen conversions in soil. Likewise, a recent study conducted by Zheng et al. (2023) through the direct extraction of soil DNA and high-throughput sequencing to investigate soil microbial communities structure in tea plantations in the Southeast region of China, reported the relative abundance of bacterial phyla corresponding to Proteobacteria (20.96–41.40%), Acidobacteria (9.41–28.42%), Firmicutes (6.39–16.03%), Bacteriodetes (6.05–13.80%), Chloroflexi (3.35–13.27%) and Actinobacteria (2.37–11.52%) being dominant phyla. Lynn et al. (2017), through the direct extraction of soil microbial DNA and 16S rRNA sequencing also demonstrated that Actinobacteria, Chloroflexi, Acidobacteria, Proteobacteria, Firmicutes dominated the diverse bacterial communities in tea plantations found in Southern region of China. Actinobacteria have evolved mechanisms such as production of secondary metabolites, production of phytohormones, production of antimicrobials and stress tolerance, enabling them to thrive successfully in various soil ecosystem including adverse conditions, this may explain their high level of occurrence. The presence of Actinobacteria in tea gardens may also be helpful for the growth and successful yield of tea plants over the years. Actinobacteria have been reported to produce enzymes and secondary metabolites including a range of antibiotics some of which have been successfully exploited commercially and industrially (Barka et al., 2016; Jose et al., 2021). They are also known to act as biopesticides for agricultural benefit and play important roles in bioremediation of chemical pesticides, heavy metals, and other toxins (Alvarez et al., 2017; Banik et al., 2019). The rich abundance of Actinobacteria in soils of tea gardens likely helps account for the ability of tea plants to resist a wide range of phytopathogens, *i.e*, through their production of antimicrobial metabolites (Shan et al., 2018).

In addition, bacteria belonging to diverse genera known as the “mycorrhizal helper bacteria” (MHB) exist in soils. Frequently, these bacteria have a tripartite association with arbuscular mycorrhizal fungi and tea roots (Bidondo et al., 2016; Gupta and Chakraborty, 2020). Although their activities in tea gardens are poorly understood, these bacterial AMF enhancers generally have been reported to promote the functions of arbuscular mycorrhizal fungi leading to a better uptake of nutrients by plants and potentially increasing their ability to survive biotic and abiotic stresses (Sangwan and Prasanna, 2022). These bacteria may also benefit the AMF which would then benefit the plant. Bacteria that function actively as mycorrhizal fungal enhancers are found in (1) the Proteobacteria, specifically within the bacteria genera: *Pseudomonas*, *Agrobacterium*, *Azospirillum*, *Azotobacter*, *Burkholderia*, *Bradyrhizobium*, *Enterobacter*, *Klebsiella* and *Rhizobium* species, (2) the Actinobacteria including *Rhodococcus*, *Streptomyces* and *Arthrobacte*r sp., and (3) the Firmicutes that include *Bacillus*, *Brevibacillus* and *Paenibacillus* sp. (Martin, 2016; Nasslahsen et al., 2022). These mycorrhizal helper bacteria may also perform several other functions not limited to enhancing AMF, such as plant growth promoting activities through the production of phytohormones (Sangwan and Prasanna, 2022), and they are widespread in tea garden soils.

### 2.3 Viral communities in soils of tea gardens

Viruses are likely the most abundant and diverse organisms on earth, many of whom affect soil microbial communities and their functions (Berliner et al., 2018; Jansson and Wu, 2022). Examination of soil viruses remains understudied; however, it is known that viruses can regulate soil microbial communities (Chevallereau et al., 2021; Liao et al., 2022) and contribute significantly to soil ecological processes such as nutrient cycling (Bi et al., 2022). In particular, there is paucity of reports on specific activities of soil viruses in tea gardens, viruses can be very important as they infect other microbial communities such as the bacteria and fungi, hence shaping microbial composition, metabolism and probably influence major soil activities (Roux and Emerson, 2022). Since viruses are host specific, viruses that infect pathogenic bacteria, fungi, and insects, have been isolated and used as biocontrol agents targeting their respective hosts (Kizheva et al., 2021). In terms of insect pests, two viruses: *Ectropis obliqua* single-nucleocapsid nucleopolyhedrovirus (EcobSNPV) and *Ectropis obliqua* picorna-like viruses (EoPV) have been commercially used with high efficacy against *E. obiqua* which is a common pest of tea plants (Idris et al., 2020). However, effective viruses for other tea pests, e.g., *Helopeltis theivora* and *Gyropsylla spegazziniana*, have not yet been commercialized, and overall, the specific contributions of soil viruses to (tea) soil fertility and plant health remains unknown.

## 3 Functions of soil microorganisms in tea gardens

**Figure 1:**
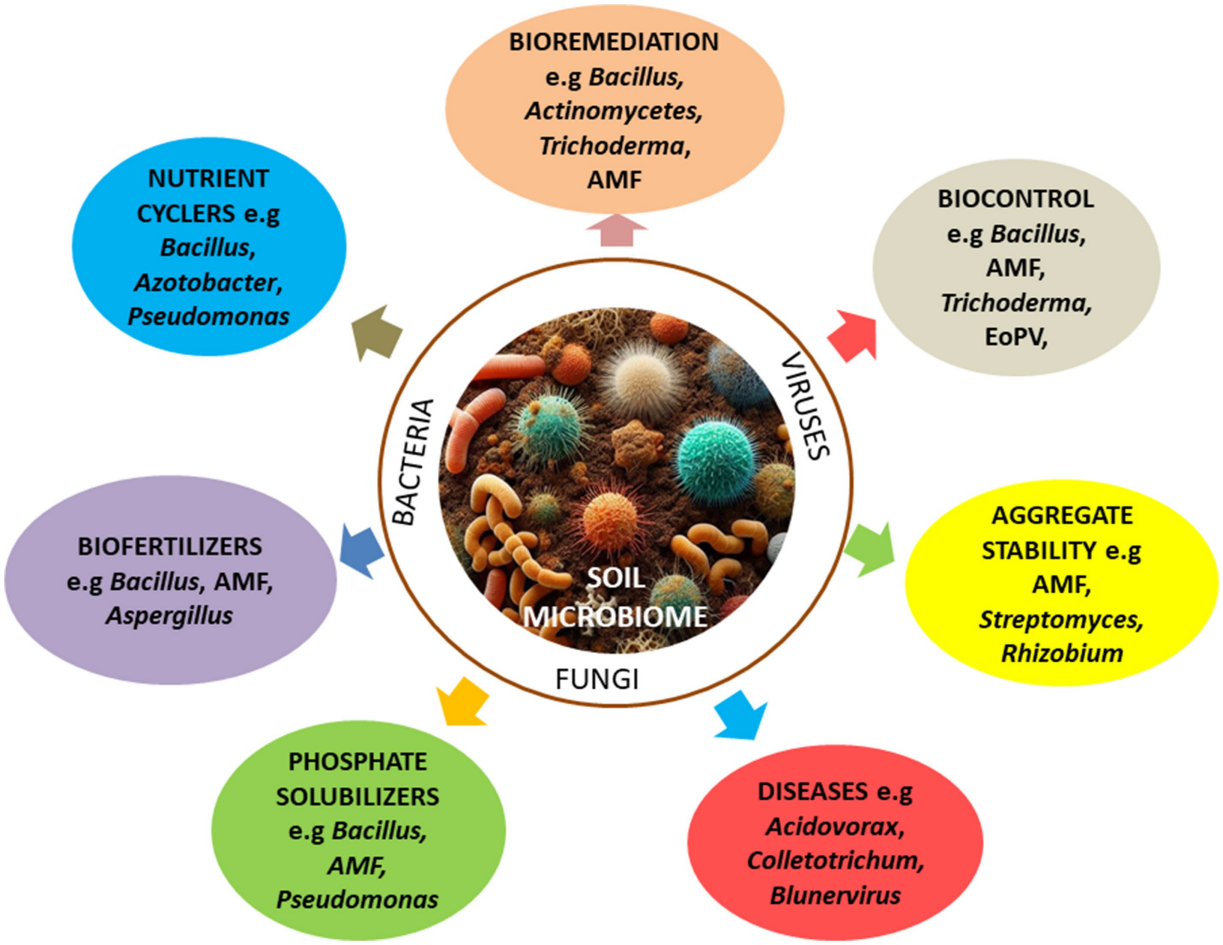
Composition and functions of soil microbial communities in tea garden.

Depending upon the member community, soil microorganisms in tea garden could have beneficial or detrimental effects on tea plants (Figure 1). Beneficial microorganisms, such as members of the *Bacillus*, *Rhizobium, Actinomycetes, Trichoderma*, and *Glomus* promote soil health and enhance plant productivity through improving soil structure and promoting organic matter recycling (Hicks et al., 2021; Wei et al., 2024), e.g., functioning as decomposers of leaf litters and dead plant materials in tea gardens (Schroeter et al., 2022; Wang et al., 2023).

Microbial communities can also help plant disease resistance, decrease soil load of (plant) pathogens, and increase (plant) environmental stress tolerances (Zhang X. et al., 2022). For example, beneficial microbes such as *Bacillus* spp. and Actinomycetes can help tea plants resist a range of fungal diseases, i.e., leaf blight and scab disease (Wang et al., 2021). Arbuscular AMF colonization in the tea rhizosphere, e.g., by *Glomus*, *Rhizophagus*, and *Acaulospora*, likely contributes to enhanced disease resistance in host tea plants. Tea roots colonization by AMF can also help tea plants to survive under adverse conditions, enhance photosynthesis, and increase nutrient (e.g., phosphorus) absorption (Bag et al., 2021; Almario et al., 2022). Conversely, some soil microorganisms, e.g., *Fusarium* and *Pseudopestalotiopsis*, can cause disease to tea plants (Arafat et al., 2020; Pandey et al., 2023), and cultivation methods such as long-term applications of chemical fertilizers (e.g., Urea- N and NPK) may result in enrichment of pathogenic fungi (e.g., *Fusarium* and *Pseudopestalotiopsis*) in tea gardens (Wang et al., 2023; Zheng et al., 2023).

Nitrosphaeraceae also play important roles in nitrogen cycling (Amoo and Babalola, 2017; Zhang Z. et al., 2022), and the rhizosphere of tea plants are frequently colonized by nitrogen fixing and ammonia oxidizing bacteria (e.g., *Azotobacter* and *Nitrosomonas*, respectively) which affect nutrient cycling in the soil and can regulate nutrient utilization in tea plants (Wright and Lehvirta-Morley, 2023). Beneficial bacteria such as *Bacillus*, *Azotobacter* and *Pseudomonas* have also been proven to be of great potential in soil remediation (Xiang et al., 2022). However, there is no information of their specific application in remediation of tea soils.

Viral lysis of microbial cells can release materials which are transformed into dissolved organic matters (Chen X. et al., 2022), thus impacting nutrient cycling processes. In addition, viruses can reprogram host metabolism by expressing virus-contained auxiliary metabolic genes during infection. These auxiliary metabolic genes are sometimes involved in numerous metabolic pathways and could boost host metabolism and supply energy, thereby enhancing viral propagation, consequently impacting biogeochemical cycles (Hurwitz and U’Ren, 2016; Bi et al., 2022). Several studies have demonstrated the presence of auxiliary metabolic genes in soils, particularly agricultural soils (Wang et al., 2016; Han et al., 2017). For example, viral-encoded carbon metabolism was identified in high organic matter peatsoils, demonstrating potential viral roles in carbon cycling processes (Emerson et al., 2018; Trubl et al., 2018). Viruses can also influence microbial/plant/animal evolution as agents of horizontal gene transfer by encoding other functional genes and mediating the transfer of genes between hosts (Trubl et al., 2018). Although assumed for many years that the tea plant was virus-free, Hao et al. (2018) reported two novel viruses belonging to the *Blunervirus* and *Ilarvirus* genera from tea plants using metagenomic analysis. These viruses infected the tea plant causing necrotic ring and discoloration of tea leaves, reducing the quality and yield of tea leaves.

## 4 Mechanisms of actions of microbial communities in tea garden soils

Some soil microorganisms’ exhibit positive plant growth promoting (PGP) traits that impact the productivity of the plants that grows on such soils (Bag et al., 2022). Growth promoting traits in soil microorganisms (e.g., *Bacillus subtilis*, *Trichoderma viridae*, and *Streptomyces griseus*) in tea garden soils (Tables 2, 3) have been shown to impact phosphate solubilization, nitrogen fixation, siderophore production, antagonism to the pathogen, and act in the production of plant auxin hormone production, e.g., indole-3-acetic acid (Bhattacharyya and Sarmah, 2018; Kolandasamy et al., 2023), with tea rhizosphere bacteria also found to promote the growth of rice and maize seedlings (Bhattacharyya et al., 2020). A significant positive relationship between Nitrososphaeraceae in tea garden soils with ammonia oxidation and nitrification processes has been reported, suggesting the importance of these bacteria in sustaining nitrogen fixation (Zhang Z. et al., 2022). These results suggest that exploitation of identified beneficial tea rhizosphere microorganisms has the potential to be used as microbial-based fertilizers. A two-year field experiment comparing the effects of bio-organic fertilizers (*Bacillus megaterium*-based bio-organic fertilizer, *Bacillus colloid*-based bio-organic fertilizer and *Bacillus subtilis*-based bio-organic fertilizer) and conventional chemical fertilizers, reported that the microbial-based fertilizers increased significantly the contents of tea polyphenols, amino acids and caffeine compared with the conventional chemical fertilizer (Liu et al., 2023), perhaps, through nitrogen metabolism and nutrient solubilization processes which enhanced nutrient availability and uptake by tea roots. Similarly, a study conducted by Xin et al. (2024) reported that the inoculation of soil with a synthetic community (SynCom21) of 21 bacterial strains belonging to the phyla Proteobacteria and Actinobacteria isolated from the rhizosphere of highly productive tea plants, was able to enhance ammonia uptake and transport in tea plants, facilitate the synthesis of theanine and increase theanine content of the tea leaves in comparison with the controls. Thus, application of tea rhizosphere bacteria as microbial-based fertilizer can promote nutrient availability and absorption resulting in enhanced tea polyphenols and theanine content, increasing the quality of tea leaves.

**Table 2:** Regulation of the functions of major soil bacterial communities in tea gardens.

Functions	Mechanisms	Phyla of microorganisms	Examples (Genera)	References
Biofertilizers (Plant growth promoters)	Indole-3- acetic acid production	Firmicutes, Proteobacteria, Actinobacteria Acidobacteria	*Bacillus, Pseudomonas, Enterobacter, Brevibacillus, Burkholderia, Leifsonia, Achromobacter, Klebsiella* *Staphylococcus, Nocardia, Ochrabactrum, Micrococcus, Arthrobacter, Streptomyces*	Dutta and Thakur (2017), Shan et al. (2018), and Bhattacharyya et al. (2020)
	Siderophore production	Firmicutes, Proteobacteria, Actinobacteria, Acidobacteria	*Bacillus, Pseudomonas, Enterobacter, Brevibacillus, Burkholderia, Leifsonia, Achromobacter, Klebsiella* *Staphylococcus, Arthrobacter, Micrococcus, Ochrabactrum, Streptomyces*	Dutta and Thakur (2017), Bhattacharyya et al. (2020), and Kolandasamy et al. (2023)
	ACC deaminase production	Actinobacteria Firmicutes, Acidobacteria Proteobacteria	*Bacillus, Pseudomonas, Enterobacter, Brevibacillus, Burkholderia, Streptomyces, Achromobacter, Klebsiella* *Staphylococcus, Ochrabactrum, Micrococcus,*	Dutta and Thakur (2017), Shan et al. (2018), Bhattacharyya et al. (2020), and Kolandasamy et al. (2023)
Biofertilizers (Nutrient cycling)	Phosphate solubilization	Firmicutes, Proteobacteria, Actinobacteria, Acidobacteria	*Bacillus, Pseudomonas, Enterobacter, Brevibacillus, Burkholderia, Arthrobacter, Achromobacter, Klebsiella* *Staphylococcus, Leifsonia, Ochrabactrum, Micrococcus, Streptomyces*	Dutta and Thakur (2017), Bhattacharyya et al. (2020), and Kolandasamy et al. (2023)
	Potassium solubilizing	Firmicutes, Proteobacteria, Acidobacteria, Actinobacteria	*Bacillus, Burkholderia, Pseudomonas, Paenibacillus, Acidothiobacillus, Rhizobium, Azospirillium, Arthrobacter*	Bagyalakshmi et al. (2017), Bhattacharyya and Sarmah (2018), and Zhang X. C. et al., 2022
	Ammonia production	Firmicutes, Proteobacteria, Actinobacteria,	*Bacillus, Pseudomonas, Enterobacter, Brevibacillus, Arthrobacter, Burkholderia, Ochrabactrum, Micrococcus, Achromobacter, Klebsiella, Leifsonia, Staphylococcus,*	Dutta and Thakur (2017) and Bhattacharyya et al. (2020)
	Nitrogen fixation	Proteobacteria, Firmicutes, Acidobacteria, Actinobacteria	*Burkholderia, Azospirillum, Pseudomonas, AcidocapsaMethylobacterium, Azotobacter, Acinetobacter, Streptomyces, Klebsiella*	Bhaduri et al. (2018), Yan et al. (2018), and Cernava et al. (2019)
Biocontrol	biosurfactant production	Actinobacteria, Firmicutes, Acidobacteria,	*Bacillus, Brevibacterium, Pseudomonas*	Banik et al. (2019) and Chopra et al. (2020a,b)
	antifungal/antibiotics production	Actinobacteria, Firmicutes Acidobacteria	*Bacillus, Pseudomonas, Enterobacter, Brevibacillus, Burkholderia, Actinomadura,Achromobacter, Klebsiella* *Staphylococcus, Serratia, Streptomyces*	Dutta and Thakur (2017), Dhar Purkayastha et al. (2018), Shan et al. (2018), and Kolandasamy et al. (2023)
Soil structure	Stabilizing soil aggregates	Actinobacteria, Firmicutes, Chloroflexi, Proteobacteria	*Streptomyces, Nocardia, Actinomadura, Rhizobium*	Wang et al. (2021) and Wang et al. (2023)
Tolerance to stress	Enhances resistance to abiotic stress	Firmicutes Actinobacteria Acidobacteria	*Pseudomonas, Bacillus, Streptomyces, Leifsonia, Ochrabactrum, Micrococcus, Arthrobacter, Nocardia, Actinomadura*	Bhattacharyya et al. (2020), Bag et al. (2021), and Kolandasamy et al. (2023)

**Table 3:** Mechanisms of the functions of major soil fungal communities in tea gardens.

Functions	Mechanisms	Phyla of Microorganisms	Examples (Genera)	References
Biofertilizers (Plant growth promoters)	Indole-3- acetic acid production	Glomeromycota Ascomycota	*Penicillium, Aspergillus, Trichoderma, AMF*	Nath et al. (2015), Chen et al. (2023), and Kolandasamy et al. (2023)
	Siderophore production	Ascomycota	*Trichoderma*	Kolandasamy et al. (2023)
	ACC deaminase production	Ascomycota	*Trichoderma*	Kolandasamy et al. (2023)
	Uptake of nutrients	Glomeromycota	*Glomus, Claroideoglomus*	Shao et al. (2018) and Li et al. (2020)
Biofertilizers (Nutrient cycling)	Phosphate Solubilizing	Glomeromycota Ascomycota	*Penicillium, Aspergillus, Fusarium, Trichoderma, Rhizophagus*	Nath et al. (2015), Bhattacharyya and Sarmah (2018), Zhang Z. et al. (2022), and Kolandasamy et al. (2023)
	Potassium solubilizing	Ascomycota, Glomeromycota,	*Penicillium, Aspergillus, Fusarium*	Nath et al. (2015)
	Ammonia production	Ascomycota	*Trichoderma*	Kolandasamy et al. (2023)
Biocontrol	Production of antibiotics/ antifungals	Ascomycota, Basidiomycota, Glomeromycota	*Trichoderma, Glomus, Rhizophagus*	Chelangat et al. (2021), Chen W. et al. (2022), and Kolandasamy et al. (2023)
Tolerance to stress	Enhances resistance of tea plant to abiotic stress	Glomeromycota	*Trichoderma, Glomus, Glomus, Clariodeoglomus, Rhizophagus*	Chelangat et al. (2021), Chen W. et al. (2021), Kolandasamy et al. (2023), and Gao et al. (2023)

Soil microbe degradation of soil pollutants is another key ecological function that entails regulated gene expression and the activities of multiple enzymes (Wang et al., 2023). A variety of chemical pesticides, especially organochlorine pesticides (OCPs) such as Dichloro-diphenyl-trichloroethanes (DDT), Endosulfan and Dicofol to target tea scale insect, mites and tea mosquito bug are routinely used in tea plantations, resulting in residues on the tea plants themselves as well as in the soil (Lu et al., 2015; Fernandes et al., 2023). These chemicals can decrease the quality of both the tea and the soil, with such persistent organic pollutants (POPs), accumulating due to their low natural degradation rates (Negrete-Bolagay et al., 2021), potentially carcinogenic (Fernandes et al., 2023). However, rhizosphere microorganisms, through the action of degradative enzymes, have been reported to be able to degrade such persistent organic pollutants (Bishnu et al., 2012; Shi et al., 2015) in the soil of tea plants. The specific microorganisms involved in the degradation of organic pollutants in tea gardens have not been reported, but tea plant root secretions such as catechin, glucose, arginine and oxalic acid, have been reported to significantly influence the degradative abilities of soil microorganisms against persistent organic pollutants (POPs) by tea plant rhizosphere microorganisms. This was explained by the reduction in the binding energy of the complex protein to POP molecules in the presence of these root secretions (Du et al., 2022). These root secretions likely also attract and stimulate select microorganisms to produce degradative enzymes such as polyphenol oxidase, hydrolases, catalase and laccase (Wei et al., 2024), which can catalyze the degradation of the POPs. Moreover, root secretions can influence microorganisms present in the rhizosphere by acting as stimulants, signaling molecules or repellants (Olanrewaju et al., 2019; Xin et al., 2024). Various plant growth promoting microorganisms including *Bacillus*, *Pseudomonas*, and *Trichoderma* have also been shown to be involved in the remediation of pollutants and heavy metals in soil (Gond et al., 2021; Ren et al., 2023). The ability of bacteria such Acidobacteria and Chloroflexi to utilize complex organic compounds has been shown to increase with the age of tea planting (Wang et al., 2020). These data indicate that metabolic activities of microorganisms in the soil of tea gardens could potentially remove and degrade harmful substances such as chemical pesticides, heavy metals and organic pollutants in the soil.

Microorganisms also help maintain soil aggregates that are important to soil structure and fertility, root penetration and crop yield, through secretions of extracellular polymeric substances and other compounds including polysaccharides, polyuronic, and amino acids with adhesive properties which can bind soil particles together (Hartmann and Six, 2022). The soil fungal community can promote aggregate stability because of their filamentous growth and their hyphal networks in soil (Morris et al., 2019). Particularly, AMF produce hyphal networks and gromalin, a putative abundantly produced glycoprotein, which aids in soil resistance to erosion, and helps increase carbon storage and water-holding capacity (Rashid et al., 2016). AMF also increase the stability of soil macroaggregates in the soil ecosystem of tea gardens (Morris et al., 2019). A report on the dynamics of soil bacterial community diversity and composition at aggregate scales in tea gardens, revealed that soil aggregates exhibited complex bacterial communities which could provide biological buffering which could prevent individual bacterial species from gaining superiority via competition or predation (Wang et al., 2021). Because stable soils can provide a valuable ecosystem for tea plants to thrive, future studies should explore the potentials of microorganisms, particularly AMF stabilization of soil ecosystem in tea gardens.

Furthermore, some soil microorganisms colonizing the root of tea plants exhibit strong biocontrol activity against plant pathogens and pests (Bag et al., 2022). Plant growth promoting fungi such as *Aspergillus*, *Fusarium, Trichoderma* and bacteria such as *Azotobacter*, *Azospirillum*, *Pseudomonas* sp., have been shown to help increase tea plant growth and can help control soil-borne plant pathogens (Thabah and Joshi, 2022), as well as potentially improving tea plant resistance to diseases (Zhang X. et al., 2022). For instance, bacterial *Bacillus* and fungal *Trichoderma* strains isolated from the tea rhizosphere have been shown to display high biocontrol efficacy against *Phomopsis theae,* a fungi pathogen causing stem canker in tea plants (Kolandasamy et al., 2023). Similarly, isolates of *B. subtilis* has been reported to significantly improve the resistance of tea against several diseases including black rot, branch canker, blister blight and root diseases (Bhattacharyya et al., 2020; Bora and Bora, 2021). Actimomycetes such as *Streptomyces*, *Microbacterium*, and *Norcardia* sp. have been reported to produce secondary metabolites with antimicrobial potentials (Shan et al., 2018) and have been proven to be successful in managing tea diseases (Bhattacharyya et al., 2016). These data indicate that healthy and/or manipulation of tea plantations soils can be useful and effective approach toward helping tea plants resist attack by microbial pathogens.

## 5 Factors that influence soil microbial communities of tea gardens

Tea is a perennial plant that is usually propagated through seedlings developed from seeds by hardening in a nursery through stepwise exposure to full daylight. Tea plant is frequently pruned to enable the development of new shoots and maintain the shape and height and can take up to two years to maturity. The leaves are harvested by plucking new leaves and terminal buds from the tip of the branches at regular intervals from the second year onwards (Mukhopadhyay and Mondal, 2017; Auria et al., 2022). The plucking of the new leaves also enables the emergence of new buds and leaves. Tea soil ecosystem functions are often affected by multiple biotic and abiotic factors. The intensity and duration of tea planting have a significant impact on the microbial community structure, biomass, and its function (Kui et al., 2021), also impacting the soil physicochemical properties.

Soil physicochemical properties like temperature, humidity and pH values influence microbial community diversity in tea garden soils (Muneer et al., 2022). Bacterial and fungal communities during tea planting are strongly affected by changes in soil pH (Zheng et al., 2023) that can occur due to the long-term use of chemical fertilizers. Soil pH in tea gardens can be altered by agricultural practices such as the addition of fertilizers and pesticides. The heavy use of chemical fertilizers can decrease soil pH while the use of organic fertilizers can regulate soil pH. Ye et al. (2022) reported that the long-term use of chemical fertilizer led to a continuous decrease in soil pH from 3.07–2.82 in tea plantations in Southeast China while the long-term use of organic fertilizer led to a stable pH of 5.13–5.33, which is suitable for growth, improved yield, and quality of tea. Persistent decreases in soil pH in addition to decreasing microbial diversity, may result in the denaturing of soil enzymes, and lowered nutrient solubility and availability to plants, as well as increased aluminium and/or heavy metals toxicity as lowered soil pH can increase solubility of certain toxic chemicals, resulting both direct plant toxicity, decreased soil microbial diversity, and equally important in terms of relevance to human consumption, accumulation of toxic metals by the plant (Yan et al., 2021; Naz et al., 2022).

Like many metals, low levels of aluminium, copper, manganese, and others are needed by the plant and promote tea plant growth, however at high concentrations coupled to lowered pH, they can induce toxicity (in the plant and/or to the consumer) as insoluble forms, e.g., for aluminium, dissociate at pH < 5 and releasing (Al^3+^) ions into the soil, which can form complexes with the other compounds (e.g., phosphates) found in the rhizosphere of tea plants (Ray et al., 2022), which would not only increase metal contents in the plants, but could also reduce the availability of phosphorus to plants. As mentioned, decreases soil pH can lead to the accumulation of metals in the tea plant leaves (de Silva et al., 2016; Peng et al., 2018; Yan et al., 2018), that when cycled, leads to a further decrease in soil pH, successively affecting soil microbial community structure and function, and ultimately impairing healthy plant growth and reducing the quality of the tea leaves. Decreased pH would favor acidophilic bacteria, i.e., those that encode genes regulating acid tolerance and/or prefer acidic conditions for their growth such as Acidobacteria (Kalam et al., 2020), and the abundance of microorganisms such as Acidobacteria and Ascomycota in tea gardens was increased with lower soil pH (Zhang Z. et al., 2022).

**Figure 2:**
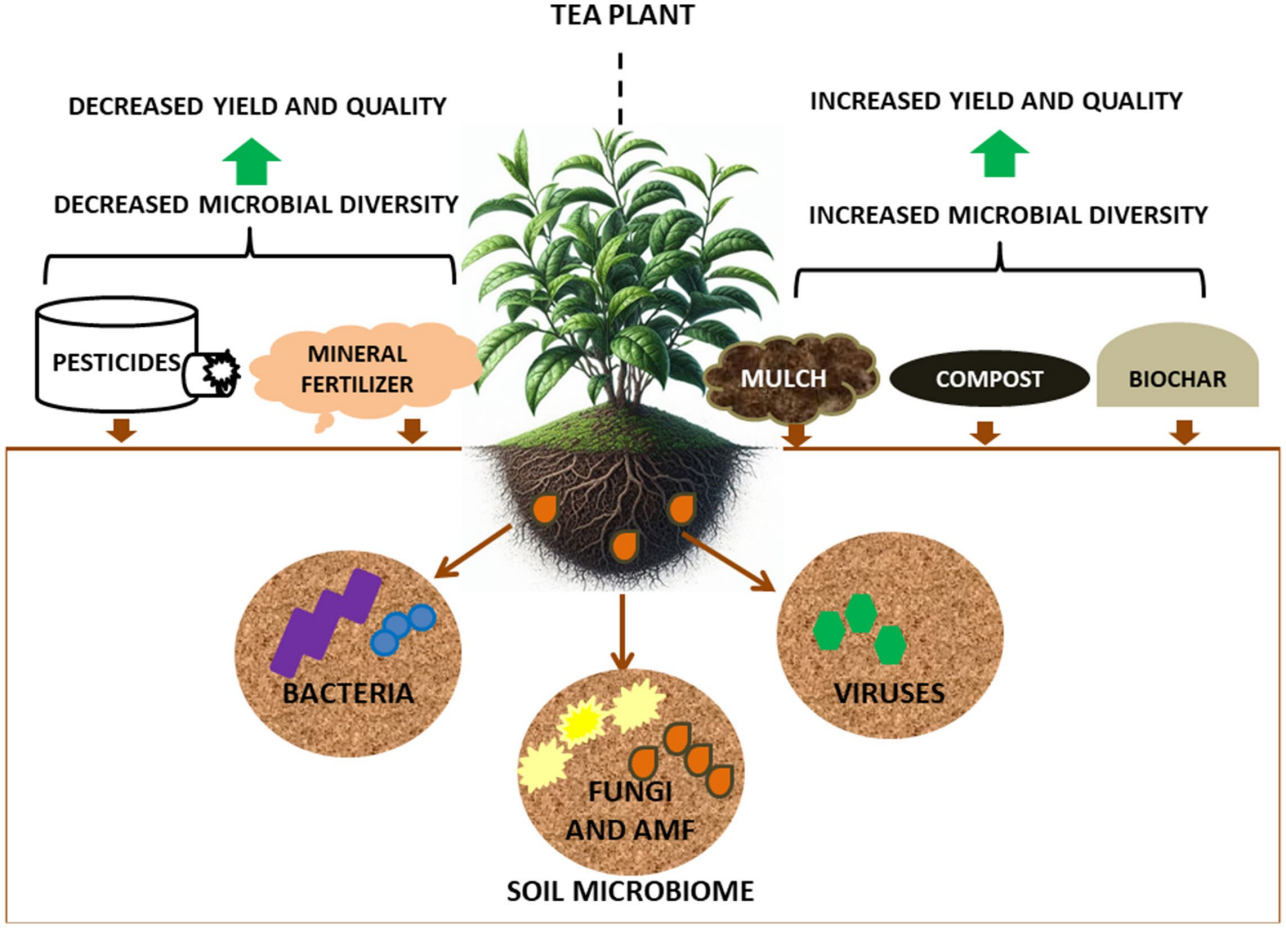
Agricultural management factors influencing microbial diversity in soils of tea gardens.

Similarly, agricultural management practices (e.g., the use of pesticides, mulch, fertilizers, Figure 2) can have beneficial or detrimental effect on the health of (beneficial) soil microbial communities, leading to increased or decreased tea plants yields, respectively. Fertilizer and pesticide applications can affect the microbial communities of the rhizosphere (Liu et al., 2021), with nitrogen fertilizers improving tea yields but leading to rapid and continuous acidification of tea garden soils (Yan et al., 2020). This acidification can result in loss of important soil microorganisms which is exacerbated by continuous tea cultivation in the same soil, which can lead to erosion of tea quality and yield (Li et al., 2016; Yang et al., 2018; Ye et al., 2022). To combat this, there has been increasing use organic fertilizers/compost as these have been to improve (i.e., help alkalinize) acidified soils, improving soil microbial community health including enzyme activities that improve soil quality (Li et al., 2018; Lin et al., 2019; Xie et al., 2021). Thus, addition of organic fertilizers to tea garden soil can be one method for the remediation of acidified soil (Ye et al., 2022). Within this context, the application of a combination of compost and nitrogen fertilizer has been shown to increase soil microbial diversity, demonstrating the compatibility of this combined approach for promoting soil and subsequent plant health (Taha et al., 2016).

A study on the effect of organic mulching in tea plantations, reported that bacteria of the phylum Nitrospirae were more numerous in peanut hull mulched soils (3.24%) as compared to polyethylene mulched soils (1.21%) (Zhang et al., 2020). The abundance of Nitrospirae indicates the presence of ammonia-and nitrate-oxidizing bacteria which are important for nitrogen cycling processes (Chen Y. P. et al., 2021). Fungal Mortierellomycota and Basidiomycota were also higher in peanut mulched soils (33.72, 21.93%) as compared to polyethylene mulched soils (14.88, 6.53%) (Zhang et al., 2020), indicating that organic mulching of tea garden soils could have a positive effect on soil microbial communities, helping to improve soil fertility for higher tea plant yields.

More recently, the biochar, which consists of carbon, volatile matter, mineral matter (ash) and moisture, created by thermal burning of biomass has been applied to soils with the aim of improving soils (Armah et al., 2023). This innovation has gained prominence as an effective soil amendment for decreasing plant disease incidence and helping promote beneficial microbial populations in continuous cropping soils (Ge et al., 2023). Biochar application to soil has been reported to increase tea plant productivity and soil nutrient contents (Zou et al., 2023). Bamboo and rice straw biochar has also been shown to significantly improve tea growth, increase tea nutrients and reduced heavy metals in tea (Yan et al., 2021). Although the mechanism of biochar mediated effects on soil microorganisms in tea gardens remains unclear, biochar application has been shown to shape the tea soil fungal community (Zheng et al., 2019), which may be because fungi play important roles in organic matter turnover (Chen et al., 2014). The mechanism of how biochar influences specific soil microbial communities for improved tea yield is an important emerging field for sustainable tea production.

## 6 Conclusions and future perspectives

Tea cultivation has considerable economic and medicinal value, and to ensure sustainable tea production, it is necessary to study the role that soil microorganism play, including promoting an increase in the diversity of beneficial soil microorganisms to improve soil health and tea productivity. Targets of future research include:

- Exploiting molecular techniques, including targeted gene manipulation (e.g., CRISPR/Cas) to enhance the beneficial characteristics of soil microorganisms, including their biofertilizing capabilities. For example, the potential of AMFs in soils (which can contribute to increased nutrient acquisition, stress tolerance and/or disease resistance) of tea plantations could be enhanced through the isolation and application of suitable strains for inoculation. In this context, molecular techniques can be used to directly manipulate tea varieties to achieve these desirable characteristics.
- Enhancing the biocontrol activity, especially toward fungal plant pathogens and insect pests, of soil bacteria and fungi in tea gardens to provide an ecologically friendly approaches disease and pest management.
- To explore and commercialize the use of plant growth-promoting microorganisms from other crops for tea cultivation and, conversely, the use of beneficial microbes derived from tea garden soils on other economically important crops for sustainable agriculture.

## Author contributions

MJ-S: Conceptualization, Methodology, Writing – original draft. ZH: Resources, Writing – original draft. NK: Writing – review & editing. YD: Data curation, Writing – original draft. RC: Data curation, Writing – original draft. SL: Data curation, Writing – original draft. YL: Data curation, Writing – original draft. PL: Data curation, Writing – original draft. JC: Data curation, Writing – original draft. CY: Data curation, Writing – original draft. WZ: Data curation, Writing – original draft. HL: Resources, Writing – original draft. ZW: Resources, Writing – original draft. SH: Resources, Writing – original draft. PC: Resources, Writing – original draft. LT: Writing – original draft. ZQ: Writing – original draft. XZ: Writing – original draft. XG: Writing – original draft. JQ: Conceptualization, Funding acquisition, Writing – review & editing.
